# Development of New Accelerated Aging Test for Comparison of the Quality of Different Insulating Papers Based on Cellulose

**DOI:** 10.3390/polym15112556

**Published:** 2023-06-01

**Authors:** Draginja Mihajlovic, Valentina Vasovic, Jelena Lukic

**Affiliations:** Laboratory for Testing and Calibration, Electrical Engineering Institute Nikola Tesla, Koste Glavinica 8a, 11000 Belgrade, Serbia; valentina.vasovic@ieent.org (V.V.); jelena.lukic@ieent.org (J.L.)

**Keywords:** aging test, Kraft paper, thermally upgraded paper

## Abstract

The aim of this study is to propose a test method for the determination of the quality of transformer paper insulation. For this purpose, the oil/cellulose insulation systems were exposed to various accelerated aging tests. The results of the aging experiments of normal Kraft and thermally upgraded papers, two different types of transformer oil (mineral and natural ester), and copper are shown. Aging was carried out in various experiments at 150 °C, 160 °C, 170 °C, and 180 °C with dry (initial values ≤ 0.5%) and moistened cellulose insulation (initial values 3–3.5%). Following insulating oil and paper, degradation markers were measured: the degree of polymerization, tensile strength, furan derivates, methanol/ethanol, acidity, interfacial tension, and dissipation factor. It was found that the aging of cellulose insulation in cycles was 1.5–1.6 times faster in comparison to continuous aging, due to the more pronounced effect of hydrolytic mechanism in cyclic aging owing to the produced and absorbed water. Furthermore, it was observed that the high initial water content in cellulose increases the aging rate two to three times more than in the dry experimental setup. The proposed aging test in cycles can be used to achieve faster aging and to compare the quality of different insulating papers.

## 1. Introduction

The life span of a transformer to a great extent depends on the loss of the mechanical properties of cellulose insulation. Due to the need to reduce the influence of high temperatures in the transformer operation, thermally upgraded (TU) papers are produced. The modification of Kraft paper by adding nitrogen-stabilized additives (melamine and dicyandiamide) is used widely to produce TU papers. The addition of these additives slows down the aging process and allows amide salts to react with the degradation product. As weak bases, they consumed water and neutralized the acidity in the oil [1,2]. The design maximum hotspot temperature inside the windings of a transformer with TU paper is higher than with normal Kraft paper [3]. On the other hand, the use of other insulating liquids (non-mineral) is increasing due to biodegradability and supposed higher temperature endurance. Ester oil has higher water solubility than mineral oil, the ability to absorb more water, and the ability to keep the cellulose insulation dry. Different types of transformer oils and cellulose insulation and their use in power transformers lead to the need to develop a standard test method for the determination of the quality of transformer insulation papers immersed in different insulating liquids. Currently, standards for the determination of the thermal class of paper-oil insulation systems are available [4,5], but these tests are complicated and long-lasting. There is no standardized procedure for the testing and comparison of the insulating papers’ quality immersed in insulating liquid. The insulating paper aging process due to the chemical reactions of hydrolysis, oxidation, and pyrolysis weaken cellulose fibers and leads to paper deploymerization and a decrease in the degree of polymerization value (DP_v_), at first, in amorphous regions, and, in advanced aging stages, in crystalline regions, causing a decrease in the mechanical strength of the paper.

Various aging tests were investigated in order to find the shortest aging time to enable the comparison of different papers’ quality. Previous studies from the literature showed the aging of different types of insulating liquids and papers in various experimental setups [1,6,7,8,9].

This investigation attempts to find a combination of parameters which can provide the aging of a paper/oil insulation system in a reasonable amount of time to achieve the end point of the DP_v_ value (250), by the combination of the hydrolytic and pyrolytic mechanisms of paper degradation, in order to avoid the application of very high aging temperatures, which are inducing artifacts in relation to the expectable highest operating temperatures in power transformers.

At aging temperatures, which are usually well above 100 °C, water is initially absorbed in the paper, migrating from cellulose through the oil and to headspace of the aging vessel, and that is the reason for the minor effect of hydrolytic degradation. In order to speed up cellulose aging, in this study, an attempt was made to investigate a way to enable the greatest possible influence of water on the aging of the paper by allowing the water to return to the paper periodically and accelerate paper aging. A comparison of the results obtained from cyclic and continuous aging experiments, the influence of applied temperatures, and other materials are given in the following chapters.

## 2. Materials and Methods

**Figure 1:**
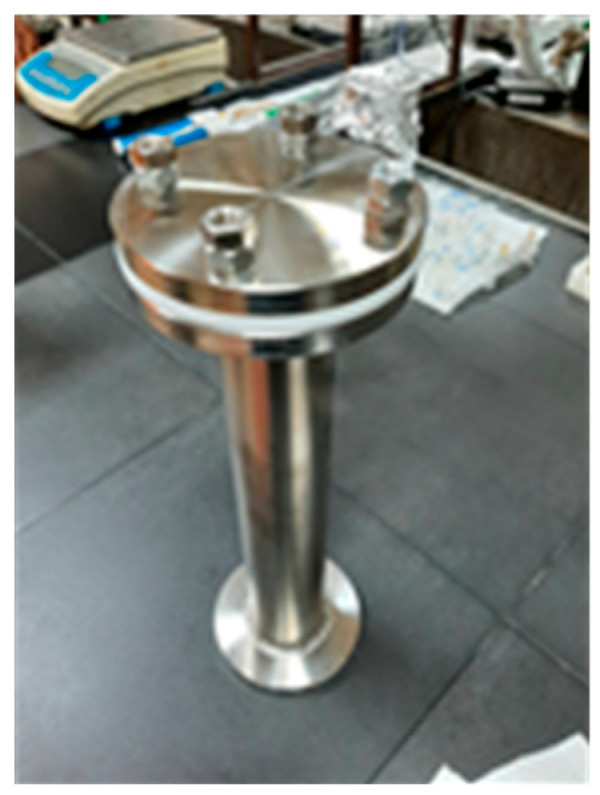
Hermetically sealed aging vessel.

Aging experiments of different types of insulating papers, pressboard, copper, and insulating oil (mineral and natural ester) were conducted in oven at various temperatures, at 150 °C for Kraft paper and, for TU papers, at 160 °C, 170 °C, and 180 °C, with dry and wet cellulose insulation. The amounts of materials were the following: oil 200 mL, 10% cellulose (paper and pressboard), and copper 400–500 cm^2^/kg oil. Aging tests were conducted in hermetically sealed stainless-steel vessels according to IEEE standard [3], in order to disable moisture loss from the paper/oil system, as this was found to be a very important factor for accelerating the degradation of cellulose insulation (Figure 1).

Preparation of insulating liquids included drying, filtering, and degassing of insulating liquid to obtain low water content prior to aging with insulating materials. Paper and pressboard were also dried to obtain low water content, below 0.5%. Drying of paper and pressboard was conducted in a vacuum chamber for 24 to 48 h at 90 °C and absolute pressure below 10 mBar, followed by impregnation with dry and degassed oil for 24 h at the same temperature and pressure. Paper strips were prepared as it is specified in the standard for tensile strength measurements (ISO 1924-3: 150 mm × 15 mm). For wet experimental setup, paper strips were subjected to wetting procedure using wet chamber at defined temperature and relative humidity for different types of oils to obtain high moisture level. After each wetting/drying procedure, papers were left to stand still, closed in flasks to further equilibrate with atmospheric temperature for 2 days.

Preparatory tests implied aging of systems made up of two different types of thermally upgraded (TU 1 and TU 2) papers, pressboard, copper, and mineral oil. Preparatory experiments were conducted for 21 and 28 days at 160 °C with purpose to compare continuous and cyclic aging. Cyclic aging was performed in duplicate using multiple 7-day heating cycles until DP_v_ ≤ 250 was reached. After each heating cycle, aging vessels were taken out of the oven to cool down for 48 h, in order to allow water migration back to the paper. After 47 h, vessels were opened for one hour to allow air ingress and to take paper samples first for measurements of water content in the paper and then for DPv determination. Paper strips sampled for DPv determination were de-oiled, dried on air, and grinded. Two solutions of grinded paper in bis(ethylenediamine)copper(II) hydroxide was made and DPv was determined according to the IEC 60450 standard [10].

These experiments were continued with aging of same types of papers in mineral and ester oils at 170 °C and 180 °C, with and without pressboard/cooper, with low and high moisture level to obtain insight in their influence on aging at defined conditions.

Further on, in order to compare aging pattern of Kraft and TU papers, cyclic aging was carried out at two temperatures: normal Kraft paper at 150 °C and TU 3 paper at 170 °C, with dry cellulose insulation (initial values ≤ 0.5% water content) and moistened papers (initial values 3–3.5% water content). Aging temperature of normal Kraft paper of 150 °C was chosen to be in the vicinity of maximum admissible temperatures in power transformer in the conditions of emergency overload (140 °C) [11]. In the case of TU papers, higher aging temperature (170 °C) was chosen based on the results obtained in preparatory tests and taking into consideration recommended ageing temperatures for expected thermal class [4] and the presence of additives. Comparison of cyclic aging pattern of normal Kraft paper and TU paper in different insulating liquids is presented.

Following parameters in oil and paper were measured: DICY index (only before aging), degree of polymerization (DP_v_), tensile strength, water content, furan derivates, methanol and ethanol, acidity, interfacial tension, and dissipation factor. Table 1, Table 2 and Table 3 show overview of aging tests, oil and paper properties, measurement equipment and used methods in different stages of aging experiments.

## 3. Results and Discussion

### 3.1. Preparatory Aging Tests—Continuous Comparing to Cyclic Aging

**Figure 2:**
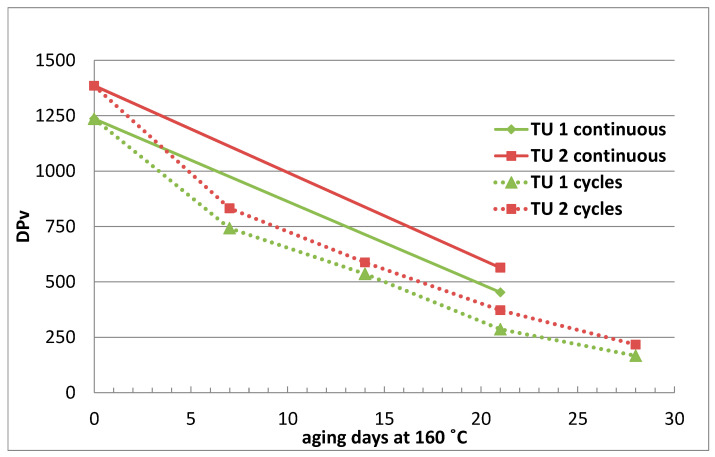
Change of DPv of TU papers during continuous and cyclic aging at 160 °C.

Preparatory aging experiments showed differences in the aging pattern of continuous vs cyclic aging with two TU papers in mineral oil at 160 °C (Figure 2).

Before and after the aging test, the Dpv value was determined, and in the case of cyclic aging, every 7 days. Cyclic aging was observed to achieve higher aging rates than continuous aging (Figure 2). After 21 days of cyclic aging, the DPv value was lower for both TU papers, and aging was faster by about 1.5–1.6 times in cyclic aging.

As it was reported in [12], the dicyandiamide (DICY) index in the TU 1 paper was 0.04 and, for TU 2, 0.15. The DICY additive slows down the degradation of cellulose by decomposition to other products [13]. In previous research [12], the same TU papers were tested in aging experiment at 140 °C with a high water content in the paper (about 3%) in four cycles, but the end DPv values in ester oil remained over 400. A slower aging rate of TU 2 compared to TU 1 was observed, owing to the higher DICY index value, indicating a higher additive content and longer protection of the paper. The higher content of nitrogen compounds is able to neutralize the aging products [2,14,15]. The DICY index was not measured during aging due to fact that it decomposed quickly as was reported earlier [12,13].

The results of the aging test at 160 °C have indicated long aging times in the case of TU papers immersed in ester insulating liquid. Therefore, in the next step, the temperature was elevated to 170 °C. TU papers immersed in mineral and natural ester oil were subjected to the same testing conditions in sealed stainless-steel vessels for 672 to 1344 h. In order to accelerate paper aging, a higher water content was introduced. The obtained results are given in Table 4.

A comparison of different system setups, i.e., dry and wet systems (initial values 0.5% and 3%) having 10% cellulose/oil ratio (10% paper only, 5% of paper, and 5% of pressboard), showed that aging was promoted by water, as expected, but was also accelerated by pressboard. This could possibly be explained by the easier migration of water absorbed in the pressboard in the system, since one previous study [16] indicated the stronger bonds of water to the paper in comparison to the pressboard. However, this effect was not observed in the case of the natural ester oil, probably due to predominant effects of water migration from the paper and pressboard, i.e., dry-out during aging in ester insulating liquid. Due to the different impact of the pressboard on the aging of cellulose/oil systems with mineral and ester oils, it was decided to continue further aging with paper only. Aging was faster in wet systems by about two times in comparison to the dry cellulose insulation/oil system. In the case of aging TU paper immersed in mineral oil, the differences in water content in the paper were evident. TU 2 paper with a higher initial DICY index had a lower water content in the paper [12].

Further on, the influence of a higher temperature was investigated by the aging of TU 1 paper in mineral and natural ester oil at 180 °C (Table 5).

The obtained DPv values implies that the aging of a dry system in mineral oil at 180 °C showed strong pyrolytic degradation, without a notable impact of the hydrolytic and oxidative mechanism. Even the impact of the metal catalyst was insignificant on TU 1 paper aging rates, both in mineral and natural ester insulating liquids.

In order to obtain a clear comparison of paper aging rates, i.e., compare different papers’ quality, keeping in mind that the pressboard is not subjected to a very high temperature in real applications but can cause different moisture migration in the paper/oil system, further aging experiments setups were prepared without the pressboard.

### 3.2. Impact of Water and Copper

The development of the testing method for the determination of the quality of thermally upgraded papers and normal Kraft insulating paper was continued as the aging tests of the paper/oil systems in cycles at two different temperatures in the presence of oxygen and copper, and water in a low and high concentration. The first experimental setup implied dry papers with initial values below 0.5%, and the second, wet papers with an initial value of 3–3.5%. The experimental setup is described in Section 2. The aging temperatures for normal Kraft paper and TU papers were different, 150 °C and 170 °C. Table 6 shows the experimental overview.

Cyclic aging was performed as described in Chapter 2. The number of cycles to reach DP_v_ ≤ 250 was between 3 and 11 for different oil/paper systems and moistness levels. Paper and oil properties were measured after every cycle and at the end of aging.

**Figure 3:**
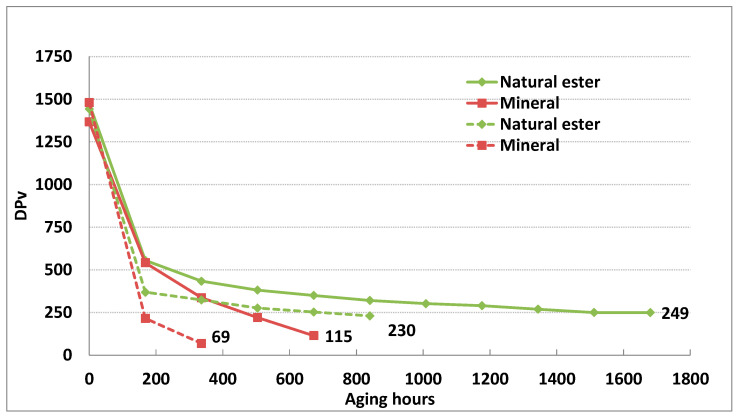
Dpv of TU3 paper during aging at 170 °C with different insulating liquids in dry and wet (dotted lines) experimental setup.

The trend of the DP_v_ change during the aging of TU 3 paper in dry and wet conditions is shown in Figure 3.

Aging was faster in the system with mineral oil, and already, after 504 h (21 days), Dp_v_ was below 250. In systems with natural ester, Dp_v_ reached values below 250 after 1344 h (56 days) of aging. At the end of the first aging cycle (168 h), Dp_v_ values had reduced by 37–40% for all systems, then a steep change was observed to continue in the case of the paper aging in mineral oil, while the paper aging rate in natural ester liquids was significantly decreased. Aging time was shorter by around 2.5 times when the paper was aged in the wet condition. After 168 h of the aging of TU paper in ester oil, DP_v_ values were below 400 and, in mineral oil, Dp_v_ values were below 250 after the first aging cycle.

Wet systems with natural ester oil age more slowly than paper immersed in mineral oil, because of the higher water solubility in ester oil, ability to absorb more water, and the ability to keep the cellulose insulation dry (Figure 3). Aging of wet TU 3 paper was faster by 2–2.2 times compared to aging in the dry condition when immersed in ester oil, while, for the same experimental setup, aging in mineral oil was up to three times faster.

**Figure 4:**
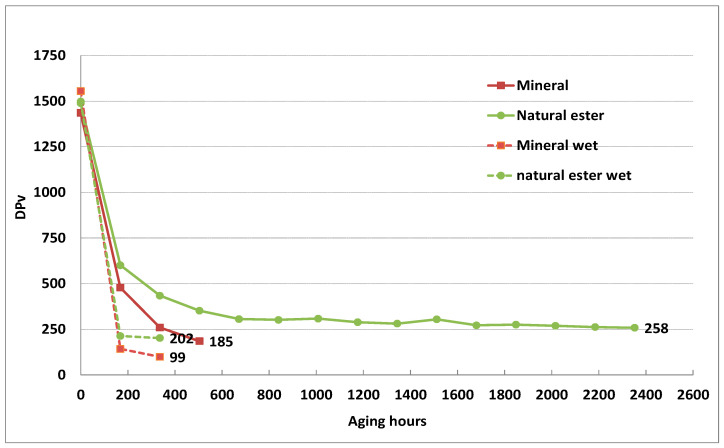
Dpv of normal Kraft paper during aging at 150 °C with different insulating liquids in dry and wet (dotted lines) experimental setup.

In the case of normal Kraft paper aging, a significant difference was observed in the aging rates of dry and wet papers immersed in natural ester (Figure 4; wet condition—dotted lines). The lowest DP_v_ value at the end was reached with mineral oil, but, after the first cycle (168 h), both paper/oil systems reached a DP_v_ below 250.

It was interesting to observe that the aging of normal Kraft paper immersed in natural ester oil at 150 °C and TU paper at 170 °C, in the dry system, had a similar trend.

The DP_v_ of aged dry Kraft paper immersed in mineral oil was 259 after 336 h (14 days) of aging, while a seven-times-longer period was needed to obtain DP_v_ values around 250 in the case of aging in natural ester oil (2352 h, 98 days). The presented results showed that ester oil extended the life of the used normal Kraft and TU paper.

**Figure 5:**
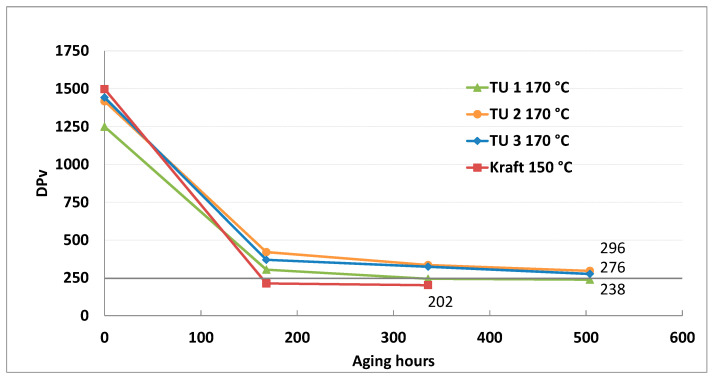
Dpv of normal Kraft and TU wet papers during aging in natural ester oil at 150 °C and 170 °C.

Figure 5 shows a comparison of the aging pattern in natural ester oil, the wet system of different TU papers at 170 °C, and Kraft paper at 150 °C.

A difference in the aging pattern is evident. TU 2 and TU 3 had a slower aging rate in the same natural ester oil. The Dp_v_ values of TU 1 and TU 2 papers remained over the 250, while TU 1 paper reached a low Dp_v_ value after the second aging cycle (336 h). This was a consequence of the lower content of nitrogen compounds in TU 1 paper, compared to TU 2 and TU 3 papers. Added nitrogen-based compounds in the papers slowed down the aging process and neutralized acids which propagate cellulose aging [2,14,15]. Aging of Kraft paper at 150 °C in the same system setup was most pronounced, and the end DP_v_ value was reached after the first cycle.

**Figure 6:**
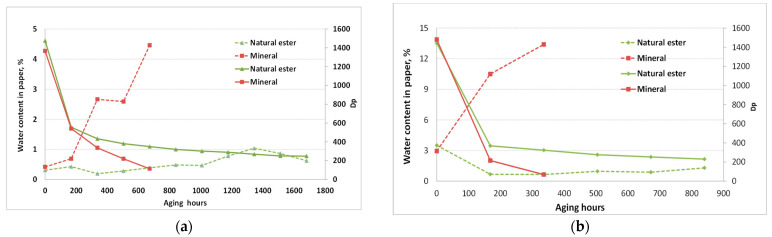
Water content (dotted lines) and Dpv of TU 3 paper during aging at 170 °C with different insulating liquids: (**a**) dry system, and (**b**) wet system.

The change of water content in paper during aging, along with DP_v_, is shown at Figure 6 (water content—dotted lines). 

**Figure 7:**
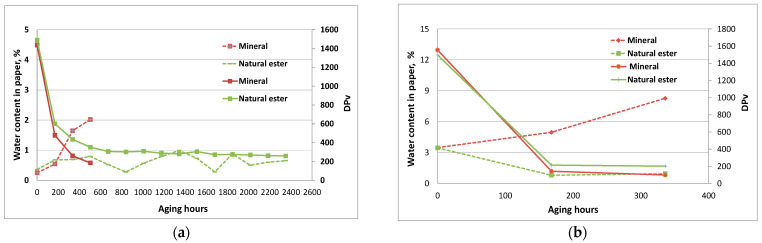
Water content (dotted lines) and Dpv of normal Kraft paper during aging at 150 °C with different insulating liquids: (**a**) dry system, and (**b**) wet system.

The increase of water content occurred in the case of paper aging immersed in mineral oil, and this was correlated with more intensive paper degradation and the production of higher amounts of water that was reflected in the step drop of the DPv value. For both the dry and wet system, the water content in TU paper at the end was much higher, by over 5% for the dry system and over 10% for the wet system when papers were aged in mineral oil. On the contrary, the water content in the paper immersed in the ester oil—wet system continuously decreased. At the end of the aging period, the wet paper water content dropped from 3% to 1.3%. Dehydration of the paper was promoted by the water consumption in ester hydrolysis and had a positive impact on slowing down the paper degradation rate. The same behavior was observed in the system with normal Kraft paper (Figure 7). 

The tensile index of paper was measured at the beginning and at the end of the aging period. The initial tensile strength for Kraft and TU papers in various oils was about 9 N/m and about 12 N/m, respectively. At the end of the aging period, the retained tensile strength of Kraft and TU 3 papers were, in natural ester oil, below 40%, and, in case of mineral oil, below 10%. The retained tensile strength was lowest for Kraft paper immersed in mineral oil. By the loss of almost all of its mechanical strength, the samples became very rigid and fragile. Low tensile indices correlated well with DPv values below 250 and the recommended criteria DPv< 250 and TS retention < 50% (25%) [4].

### 3.3. Paper/Oil Aging Markers

Oil and paper aging markers were measured at the beginning and at the end of the aging period in order to additionally obtain insight in the paper/oil functional properties after aging. Paper aging markers, such as furan derivates, methanol, and ethanol, were measured, as well as oil aging markers: acidity, interfacial tension, and dissipation factor (Table 7).

Significant differences between oils were observed in almost all investigated properties. The acid content in ester oils was much higher than in mineral oil, due to the hydrolysis reactions of ester during aging. This was pronounced in the case of Kraft paper aging. However, high concentrations of fatty acids produced from natural ester oil hydrolysis did not have a strong effect on paper degradation [17]. The acid content in ester oil—wet condition, were similar regardless of the type of aged paper.

The concentrations of furans were 10 times lower in ester oil than in mineral oil. This is possibly owing to their consumption in the reaction with nitrogen compounds in the TU paper [18], while, in Kraft paper, this is possibly owing to the effect of the stronger bonding of ester groups to cellulose OH groups, causing the restricted dissolution of furans in ester oil [15]. A significant concentration of furan derivates was dissolved by aging TU paper in mineral oil. Methanol concentrations were much lower in ester oil, probably as a consequence of the reaction of methanol with acids [7,17]. The same was observed for ethanol concentrations. Dissipation factor values in ester oil were very high, unlike in mineral oil, owing to the much higher polarity of ester oils than mineral oils [19,20,21].

## 4. Conclusions

The proposed new accelerated aging tests in cycles was performed in order to investigate the impact of water generated and absorbed in the paper on the increase of paper aging rates, imitating the cyclic mode of transformer loading with the attempt to mimic the effects of pyrolysis, oxidation, and hydrolysis. Cyclic aging consisted of a repetition of the application of high elevated temperatures, followed by cooling down and resting at ambient temperatures in hermetically sealed vessels. It was found that this test accelerated the paper aging process for the same experimental setup in continuous aging, due to the more pronounced effect of the hydrolytic aging mechanism in cyclic aging owing to the produced and absorbed water.

The presented experimental results showed that cyclic aging increased the aging rate for 1.5 to 1.6 times in comparison to continuous aging. The reported results showed that ester oil extended the life of the examined normal Kraft and TU papers. High initial water contents in the paper were found to increase aging rate by two to three times, causing a shortening of aging times, but also decreased the difference in aging times of the same paper immersed in different oils.

It was found that the proposed aging test differentiates well between the quality of the investigated insulating papers. Further work should be focused on the application and further development of the proposed accelerated aging tests on different types of insulating papers, within working group CIGRE WG D1.76 “Tests for verification of quality and aging performance of cellulose insulation for power transformers”.

## Figures and Tables

**Table 1:** Overview of aging tests.

Insulating Paper	T, °C			
	150	160	170	180
Thermally upgraded, TU 1		continuous/cycle	cycle	cycle
Thermally upgraded, TU 2		continuous/cycle	cycle	
Thermally upgraded, TU 3			cycle	
Kraft	cycle			

**Table 2:** Oil and paper properties and used equipment and methods.

Properties	Measurement Equipment	Method	Before Aging	After Every Cycle, 7 Days	At the End of Aging
Water content in paper	Methrom 737 KF Coulometer with 832 KF Thermoprep	IEC 60814	x	x	x
DPv of the cellulose materials	Viscometer tube	IEC 60450	x	x	x
Tensile strength	EZ-LX Test Shimadzu	ISO 1924-3	x		x
Dielectric dissipation factor	Baur Oil Tester DTL C	IEC 60247	x		x
Interfacial tension	Krus GMbH model K11	ASTM D971-12	x		x
Acidity	Colorimetric titration	IEC 62021-1	x		x
Water content in oil	Methrom 831 KF Coulometer	IEC 60814	x		x
Furan	Thermo Scientific DIONEX Ultimate 3000	IEC 61198	x		x
Methanol/Ethanol	Agilent 7890B TOGA	IEC TR 63025	x		x
DICY index	FT-IR Nicolet iS10	FTIR-ATR in-house	x		

**Table 3:** Insulating papers’ properties.

Insulating Papers	Thickness, mm	DICY Index
Kraft	0.06–0.125	/
Thermally upgraded, TU 1	0.06–0.125	0.04 [12]
Thermally upgraded, TU 2	0.06–0.125	0.15 [12]
Thermally upgraded, TU 3	0.06–0.125	0.02

**Table 4:** Water content and DPv value of paper and pressboard in dry/wet system at 170 °C with 10% cellulose/oil ratio (5% paper + 5% pressboard, 10% paper).

Insulating Oil	Insulating Paper	Aging Hours	Dry System Paper and Pressboard			Wet System Paper and Pressboard			Wet System Paper Only
			WC Paper, %	WC Pressboard, %	DPv	WC Paper, %	WC Pressboard, %	DPv	DPv
Mineral	TU 1	initial	0.35	0.11	1236	3.32	3.10	1236	1236
		192	2.00	1.10	426	8.93	8.43	94	236
		336	1.74	0.60	220				118
		504	3.66	2.06	147				
		672	12.26	4.67	75				
	TU 2	initial	0.44	0.11	1385	3.14	3.10	1385	
		192	1.45	0.70	557	5.00	6.00	152	
		336	0.21	0.24	417				
		504	1.44	0.65	318				
		672	3.51	1.73	192				
Natural ester	TU 1	initial	0.41	0.13	1249	3.56	3.37	1249	1249
		192	0.97	0.23	516	0.57	0.07	353	304
		336	0.33	0.11	385	0.92	0.22	312	244
		504	0.64	0.15	326	0.7	0.39	239	238
		672	0.33	0.19	260	0.94	0.24	209	
		840	0.93	0.24	239	1.1	0.42		
		1008	0.43	0.32	208				
	TU 2	initial	0.41	0.13	1417	3.40	3.37	1417	1417
		192	0.78	0.18	547	0.60	0.20	427	420
		336	0.28	0.13	420	0.68	0.29	314	335
		504	0.23	0.24	372	0.53	0.24	272	296
		672	0.15	0.15	322			256	
		840	0.37	0.16	308				
		1008	0.45	0.14	294				
		1176	0.84	0.17	267				
		1344	1.28	0.25	239				

WC—water content, %.

**Table 5:** DPv values of paper in systems: paper only and paper/copper at 180 °C.

Insulating Paper	Insulating Oil	Aging Hours	DPv	
			Paper Only	Paper and Copper
TU 1	Mineral	0	1236	1236
		192	299	281
		336	167	146
	Natural ester	0	1249	1249
		192	364	378
		336	299	300
		504	266	249
		672	237	238
		840	210	218

**Table 6:** Experimental overview.

Insulating Oil	Insulating Paper and Aging Temperature	
	Normal Kraft, 150 °C	Thermally Upgraded, TU 3, 170 °C
Mineral	dry/wet	dry/wet
Natural ester	dry/wet	dry/wet

**Table 7:** Oil properties after aging tests.

Insulating Paper	Experimental Setup	Insulating Oil	Aging Hours	Oil Properties					
				Furans mg/kg	MetOH µg/kg	EtOH µg/kg	Acidity, mg_KOH_/g_oil_	Interfacial Tension, mN/m	Dissipation Factor, ‰
TU 3 170 °C	dry	NE	1680	2.3	7	62	13.2	18	1540
		MO	672	12.04	6863	873	0.305	28	7.295
	wet	NE	840	3.73	92	61	21	13	3196
		MO	336	30.2	3955	190	0.44	27	3.18
Kraft 150 °C	dry	NE	2352	10.61	35	3	9.3	18	569.1
		MO	504	83.06	3673	908	0.09	22	5.48
	wet	NE	336	6.49	102	8	20.3	15	251
		MO	336	183.92	1778	438	0.28	31	2.295

Legend: NE—natural ester, MO—mineral oil.

## Data Availability

The data presented in this study are available on request from the corresponding author.
